# Modification of Mechanical and Electromechanical Resonances of Cellular Ferroelectret Films Depending on the External Load

**DOI:** 10.3390/polym13193239

**Published:** 2021-09-24

**Authors:** Julio Quirce Aguilar, Tomás Gómez Álvarez-Arenas

**Affiliations:** ITEFI-CSIC, Serrano 144, 28006 Madrid, Spain; j.quirce@csic.es

**Keywords:** cellular polymers, ferroelectrets, thickness resonances, piezoelectric polymers, ultrasonic transduction

## Abstract

Ferroelectret films are cellular polymers with electrically charged pores that exhibit piezoelectric response. Among other applications, ferroelectret films have been widely used as active elements in air-coupled ultrasonic transducers. More recently, they have also been tested in water immersion. They show a promising wide frequency band response, but a poor sensitivity produced by the disappearance of the electromechanical resonances. This paper studies in detail the modification of FE films response when put into water immersion, both the mechanical and the electromechanical responses (the latter in transmission and reception modes). The lack of electromechanical thickness resonances when the films are put into water is explained as the result of the different profile of the modification of the polarization vector along the film thickness imposed by the large mechanical load produced by the water. This different electromechanical response can also be the reason for the subtle modification of the mechanical thickness resonances that is also observed and analyzed.

## 1. Introduction

Ferroelectrets are cellular polymer films that contain flattened and elongated pores in the film plane with the capability that these pores can be electrically charged in a stable way [1,2]. Cellular porous solids are widely found in nature as this is a kind of hierarchical structure that provides many different design advantages and offer the possibility of combining different functionalities. They are also commonly found in medicine, both as organs that need to be studied and as a source of inspiration for the design of sensors. [3]. In addition, programmable materials based on cellular solids have also been proposed to recreate the essential features of biologically self-adaptive materials [4]. As a result of their cellular structure, ferroelectrets are thin and flexible polymeric films that exhibit piezoelectric properties. Different manufacturing techniques have been used, such as the two-step inflation technique [5], template-patterning techniques [6,7] and additive manufacturing techniques [8]. This cellular structure is designed so that the deformation of the material takes place through bending of the pore walls and the possibility of trapping electrical charge in the pores in a stable way is maximized [9]. This gives rise to very reduced elastic modulus, so relatively large deformations can be achieved. This feature together with the trapped electrical charge in the pore walls gives rise to a macroscopic piezoelectric response. Previous studies of ferroelectret materials have been oriented towards characterizing the films, modifying the cellular microstructure to maximize the piezoelectric response and stabilizing and optimizing the electrical charge trapped in the pores [5,10,11,12,13,14,15,16]. More recently, a new type of material combining conventional piezoelectricity (linked to microscopic charge distribution) and ferroelectret piezoelectric response (linked to macroscopic electrical dipoles trapped in the macroscopic pores) has been proposed and used as a biometric sensor [17].

Ferroelectrets have been used for many different applications including microphones [18], energy harvesting [19,20,21], wearable devices [22] and flexible and printable sensors (FLEPS) [23], flexible touch pads and tactile sensors [24], and air-coupled ultrasonic transducers [25,26,27,28,29,30,31,32,33]. In the latter case, the main advantage is the extremely low acoustic impedance of these materials that facilitates the coupling to the air; they are normally operated using the thickness resonances of the films. In addition, the possibility to operate these films in the electrostrictive regime under high voltage excitation offers an important improvement of transducer efficiency [34,35]. Applications of FE-based air-coupled transducers have mainly been oriented towards non-destructive testing of materials. More recently, ferroelectret films have also been revealed as a promising candidate to produce transducers for liquid coupling and hydrophones. Applications in the low frequency range (below 100 kHz) have been studied in [36,37,38] while applications for wideband ultrasonic transducer for medical applications and hydrophones, involving a frequency range >200 kHz, have been proposed and studied in [39,40,41]. They present an extremely wideband response but a very low sensitivity compared with conventional transducers. In addition, they also present some abnormalities compared with the response of FE transducers for air-coupled ultrasonic applications that should be better understood in order to be able to improve their performance in water immersion applications—in particular, to improve sensitivity without compromising the bandwidth and to compensate the sensitivity loss when frequency increases.

Ref. [42] supposed a step forward, as it showed that air-loaded thickness resonances of these films can be better explained if a sandwich meso-structure together with a cellular microstructure is assumed. This conclusion is consistent with SEM images of the FE film structure. This approach permitted to justify the harmonic distortion of the thickness resonance spectra observed in these films.

The purpose of this paper is to study, simultaneously, both thickness resonances and electromechanical response (both as receiver and transmitter) of this type of material as well as the modifications in both of them when going from the well-known case of air-loaded films to the more unconventional case of water-loaded films, which is of interest for medical transducers and hydrophones, as suggested above. Given that the impedance of these materials is about 0.05 MRayl and impedance of water and air are 1.5 MRayl and 4 × 10^−4^ MRayl, respectively, the variation of boundary conditions when the film is in water or in air is remarkable.

Towards this end, two different FE films have been studied both in air and in water. First, we measured the transmission coefficient in water immersion at normal incidence and for a frequency band that, at least, covers the first order of the thickness resonances—in most cases, the first two orders. Similar measurements were performed in air for the same materials [15,42]; in the latter case, material parameters were extracted by assuming a sandwich structure for the film and solving the inverse problem. Observed resonances in air and in water have been compared and differences have been analyzed.

Then, the electromechanical response of the films is measured, both under air and water loads. Two different types of measurements were performed in this case. The first one consists of measuring the generated electric signal in the FE when an ultrasonic signal impinges on the film at normal incidence, while the second one consists of measuring the radiated ultrasonic signal when an electrical excitation is applied to the film (Tx mode). These measurements were performed both in water and in air. Responses of the film under these two different loading conditions are compared. Finally, an explanation for the observed differences is provided.

## 2. Materials and Methods

### 2.1. Materials

Two different ferroelectret (FE) films have been used for this study, both from EMFIT Ltd (Vaajakoski, FINLAND), commercial names: HS03 and HS06. Properties of these films can be seen in Table 1 and in Refs. [15,41,42].

**Figure 1:**
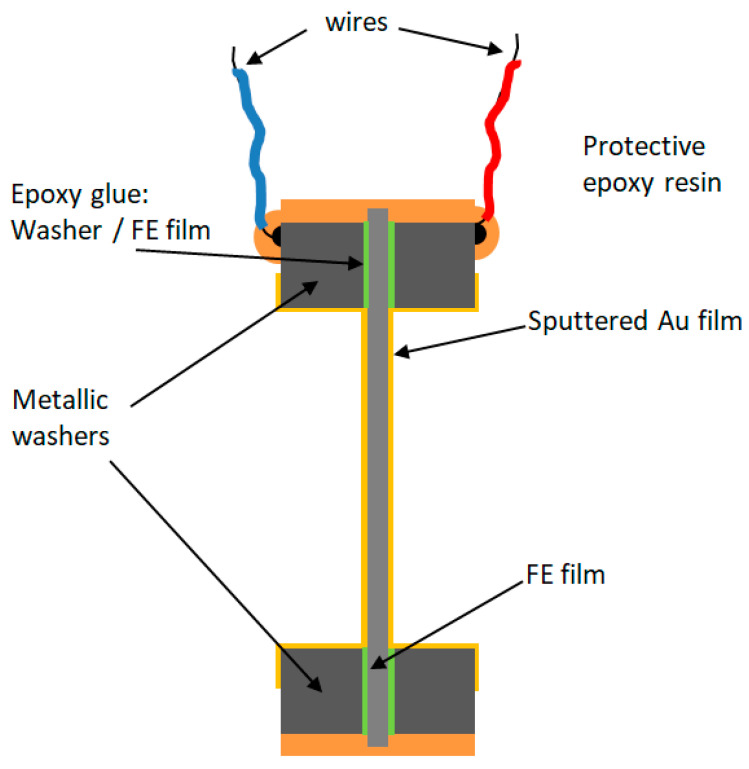
Schematic representation of the FE film preparation.

To facilitate sample handling and electrical connections for the measurement of the electromechanical response, samples were prepared by sandwiching the FE film between two metallic washers (outer diameter: 40 mm, inner diameter: 25 mm). The FE and the washers were glued using epoxy resin. Once glued, both free surfaces of the FE film and washer were Au sputtered (using a LEICA EM ACE200 sputtering LEICA, Wetzlar, Germany), for 60 s) to ensure electrical conductivity between the washer and the surface of the FE film the washer is glued to. Finally, two wires were soldered to the washers and epoxy resin was applied to the edge of the washers and to cover the soldering points as protection and to facilitate the handling of the samples. The structure and composition of the samples so prepared is shown in Figure 1.

**Figure 2:**
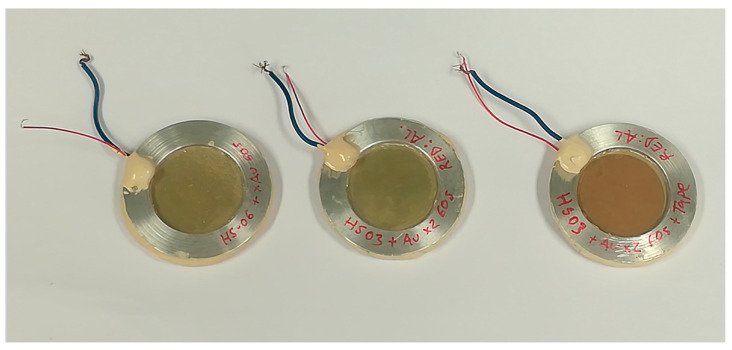
Picture of the prepared FE films for measurements. The HS03 sample with the adhesive tape is shown on the right; the brawn adhesive tape can be seen on top of the Au layer.

The samples resemble tambourines (see pictures in Figure 2). Two samples using HS03 film and one sample using HS06 film were prepared. In one of the HS03 samples, a film of adhesive tape (150 μm thick) was glued to one of the faces of the FE film. Due to the very small thickness and impedance of the FE film (0.046 MRayl), the presence of this adhesive tape film is “seen” as very high impedance load (impedance of the adhesive tape ~1.7 MRayl). Hence, the observed thickness resonances of this sample in air are shifted closer to the quarter wavelength resonances, while for the other samples, we observe the half wavelength resonances of the free-standing film. On the contrary, when submerged in water, as the impedance of the water is very close to the impedance of the adhesive tape, the response of the FE film with the adhesive tape is expected to be very similar to that of the free film (without adhesive tape). 

**Figure 3:**
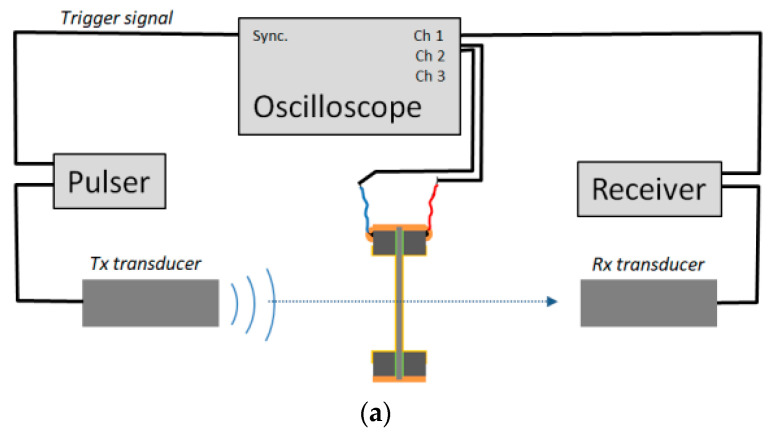

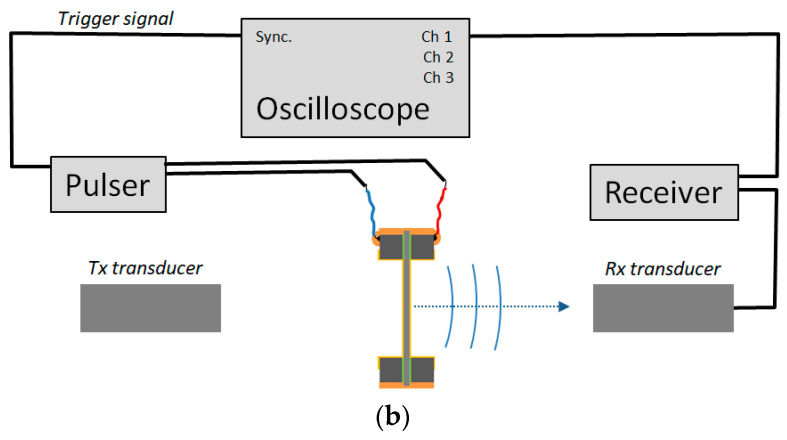
Experimental set-up. (**a**) Configuration for the measurement of both the transmission coefficient and the FE film and the film response in Rx mode; (**b**) configuration for the measurement of the FE film response in Tx mode.

The experimental set-up is shown in Figure 3. A couple of identical ultrasonic transducers are positioned in opposition and aligned and the sample to be measured is located in between them at normal incidence.

**Figure 4:**
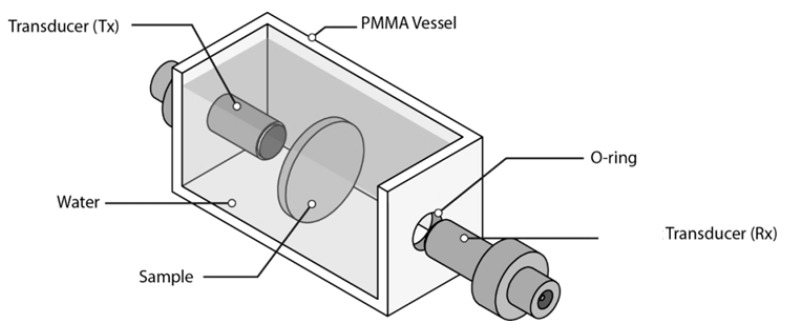
Water tank for water immersion measurements.

Two different media, where both sample and transducers are immersed, have been used: water and air. When water immersion is used, a small water tank is used as shown in Figure 4.

All measurements were performed at room conditions. For water immersion measurements, a pair of wide band transducers (Olympus, Olympus NDT Inc., Quebec, Canada, Ref #V303, 15 mm diameter, 1 MHz center frequency) have been employed. These transducers permit to cover the frequency range from 0.2 to 1.4 MHz. In some cases, it was of interest to expand the frequency range to higher frequencies. In these cases, a second pair of transducers, also from Olympus (Olympus NDT Inc., Quebec, Canada), centered at 2.25 MHz were used (15 mm diameter, Ref #C306). This permitted to expand the frequency range up to 3.0 MHz.

For air-coupled measurements, three pairs of air-coupled transducers manufactured at ITEFI-CSIC (Madrid, Spain) were used to cover a similar frequency range. The centre frequency of these three pairs of transducers is: 0.25, 0.65 and 1.1 MHz, respectively.

In all cases, the transmitter transducer was driven by using an Olympus pulser/receiver (5058PR), *pulser* in Figure 3. The same pulser was also used when the FE film was excited to measure its response in transmission (Tx) mode, as shown in Figure 3b. This pulser generates a wideband spike. Amplitude of the excitation was set to 200 V for air-coupled measurements and to 100 V for water immersion measurements. Gain in reception stage of the 5058 P/R (*receiver* in Figure 3) was between 0 and 10 dB for water immersion measurements and between 10 and 20 dB for air-coupled measurements; all filters in the receiver (5058PR) were off.

Without sample in between transducers, the fast Fourier transform (FFT) of the signal received in the scope ($FFT\left(S_{ref}\right)$) can be used to characterize the response of the system. $FFT\left(S_{ref}\right)$ is the result of the multiplication of the transfer functions (in frequency domain), *TF*, of the different elements present in the experimental set-up (i.e., *pulser*: electrical excitation, *Tx*: transmitter transducer, *Rx*: receiver transducer, *receiver*: electronics at reception, i.e., gain, matching impedance, etc.), and applying them to the input of the system: the signal provided by the pulser (${\left[S_{pulser}\right]}^{*}$):

(1) $$FFT\left(S_{ref}\right)=TF\left(receiver\right)\times TF\left(Rx\right)\times TF\left(fluid-gap\right)\times TF\left(Tx\right)\times {\left[S_{pulser}\right]}^{*}$$

Alternatively:

(2) $$FFT\left(S_{ref}\right)=TF\left(receiver\right)\times TF\left(fluid-gap\right)\times TF{\left(Tr\right)}^{2}\times {\left[S_{pulser}\right]}^{*},$$

where $TF{\left(Tr\right)}^{2}=TF\left(Tx\right)\times TF\left(Rx\right)$.

In addition, TF(receiver) can be split into two terms:

(3) $$TF\left(receiver\right)=G\times TF\left(receiver^{*}\right),$$

where *G* is the gain in reception and $TF\left(receiver^{*}\right)$ is the result of the electrical impedance matching between receiver transducer (*Rx*) and the electronics in the receiver.

In some cases, Equation (2) can be further simplified. For example, for wide band transducers TF(Tx) ≈ TF(Rx). Under spike excitation and for pulser bandwidth much larger than transducers bandwidth, it can be assumed that ${\left[S_{pulser}\right]}^{*}$ ≈ cte = A, at least within the transducer’s frequency band. Finally, for a receiver with flat frequency response, *G* in Equation (2), can be considered cte. Then Equation (3) can be simplified:

(4) $$FFT\left(S_{ref}\right)=A\times G\times TF{\left(Tr\right)}^{2}\times TF\left(fluid-gap\right)\times TF\left(receiver^{*}\right),$$

Three different measurements were performed for all of them the sample remained in the same position and the pulser configuration; that is, ${\left[S_{pulser}\right]}^{*}$ was also kept unchanged:

1. the transmission coefficient of the FE sample,
2. the electrical voltage generated in the FE sample when an ultrasonic wave impinges on the it (electromechanical response in *Rx* mode),
3. the ultrasonic signal emitted by the FE sample when an electrical excitation is applied to it (electromechanical response in *Tx* mode).

### 2.2. Methods

#### 2.2.1. Measurement of the Mechanical Response of the FE Sample: Transmission Coefficient

As explained before, the system is characterized by measuring $FFT\left(S_{ref}\right)$. Then, the FE sample is put in between *Tx* and *Rx* transducers at normal incidence. All elements of the system remain unchanged with the exception of the gain in the receiver, which is increased. As in the previous case, the FFT of the signal in the receiver transducer, $FFT\left(S_{sample}\right)$, is the result of the multiplication of the transfer functions (in frequency domain) of the different elements in the experimental set-up applied to the FFT of the input signal, which is the signal provided by the pulser: ${\left[S_{pulser}\right]}^{*}$:

(5) $$\begin{array}{cc} FFT\left(S_{sample}\right)= & TF\left(receiver\right)\times TF\left(Rx\right)\times TF\left(fluid-gap\;2\right)\times TF\left(sample\right)\times TF\left(fluid-gap\;1\right)\\ & \times \;TF\left(Tx\right)\times {\left[S_{pulser}\right]}^{*} \end{array}$$

For films with thickness << distance between *Tx* and *Rx*, it can be assumed that:

(6) TF(fluid−gap 1)TF(fluid−gap 2)=TF(fluid−gap),

So, Equations (2), (5) and (6) lead to:

(7) $$FFT\left(S_{sample}\right)=TF\left(sample\right)FFT\left(S_{ref}\right),$$

then:

(8) $$TF\left(sample\right)=\frac{FFT\left(S_{sample}\right)}{FFT\left(S_{ref}\right)},$$

and TF(sample) is equal to the transmission coefficient of the film, the modulus (in dB) is obtained from:

(9) $$20log\left|\frac{FFT\left(S_{sample}\right)}{FFT\left(S_{ref}\right)}\right|,$$

#### 2.2.2. Measurement of the Electromechanical Response: Rx Mode

Keeping the same experimental configuration, we measured the electromechanical response of the film in receiver mode. Following Figure 3a, this is obtained by measuring the FFT of the signal at channel 2 of the scope (i.e., the electrical voltage generated in the FE sample when the ultrasonic signal generated by *Tx* impinges on it).

The FFT of this signal,$\;FFT\left(S_{FE-RX}\right)$, is given by:

(10) $$FFT\left(S_{FE-RX}\right)=TF\left(receiver\right)\times TF\left(FE_{Rx}\right)\times TF\left(fluid-gap\;1\right)\times TF\left(Tx\right)\times {\left[S_{pulser}\right]}^{*}$$

Moreover, if the sample is located in the middle of the fluid-gap:

(11) $$TF\left(fluid-gap\;1\right)=TF\left(fluid-gap\;2\right)\approx TF{\left(fluid-gap\right)}^{1/2},$$

Then Equation (4) is:

(12) $$FFT\left(S_{ref}\right)=A\times G\times TF{\left(Tr\right)}^{2}\times TF\left(fluid-gap\right)\times TF\left(receiver^{*}\right),$$

Then, with Equations (4) and (11), Equation (10) can be written as:

(13) $$FFT\left(S_{FE-RX}\right)=A\times G\times TF\left(Tr\right)\times TF{\left(fluid-gap\right)}^{1/2}\times TF\left(FE_{Rx}\right)\times TF\left(receiver^{*}\right)$$

Then:

(14) $$TF\left(FE_{Rx}\right)=\left(FFT\left(S_{FE-Rx}\right)/\sqrt{FFT\left(S_{ref}\right)}\right)\times 1/\sqrt{A\times G\times TF\left(receiver^{*}\right)},$$

That is:

(15) $$TF\left(FE_{Rx}\right)\propto FFT\left(S_{FE-Rx}\right)/FFT{\left(S_{ref}\right)}^{1/2},$$

and the modulus of $TF\left(FE_{Rx}\right)$ in dB is given by:

(16) $$20log\left|TF\left(FE_{Rx}\right)\right|=20log\left|\frac{FFT\left(S_{FE-Rx}\right)}{FFT{\left(S_{ref}\right)}^{1/2}}\right|-cte,$$

where: $cte=10log\left(A\times G\times TF\left(receiver^{*}\right)\right)$

The magnitude: $20log\left|TF\left(FE_{Rx}\right)\right|+cte$ is defined as: $20log\left|TF{\left(FE_{Rx}\right)}^{*}\right|$. This is straightforwardly obtained from the measurements and represents the frequency profile of the FE sample response in Rx mode. Where $S_{FE-Rx}$ is the electric voltage measured at FE film terminals. In this configuration, the electrical voltage in the FE,$\;FFT\left(S_{FE-RX}\right)$, is produced by the ultrasonic signal transmitted by the *Tx* transducer and the piezoelectric effect of the FE film.

#### 2.2.3. Measurement of the Electromechanical Response: Tx Mode

Finally, we measured the electromechanical response of the film in transmission mode: $FFT\left(S_{FE-TX}\right)$. Towards this end, the pulser output is connected to the FE film wires and the signal received at the receiver transducer (channel 1 of the scope in Figure 3b) is registered.

(17) $$FFT\left(S_{FE-TX}\right)=TF\left(receiver\right)\times TF\left(Tr\right)\times TF\left(fluid-gap\;1\;\right)\times TF\left(FE_{Tx}\right)\times {\left[S_{pulser}\right]}^{*},$$

or:

(18) $$FFT\left(S_{FE-TX}\right)=A\times G\times TF\left(FE_{Tx}\right)\times TF{\left(fluid-gap\right)}^{1/2}\times TF\left(Tr\right).$$

That is:

(19) $$TF\left(FE_{Tx}\right)=\left(FFT\left(S_{FE-Tx}\right)/\sqrt{FFT\left(S_{ref}\right)}\right)\times 1/\sqrt{A\times G\times TF\left(receiver^{*}\right)},$$

and the modulus of $TF\left(FE_{Tx}\right)$ in dB is given by:

(20) $$TF\left(FE_{Tx}\right)\propto FFT\left(S_{FE-Tx}\right)/FFT{\left(S_{ref}\right)}^{1/2},$$

and the modulus of $TF\left(FE_{Tx}\right)$ in dB is given by:

(21) $$20log\left|TF\left(FE_{Tx}\right)\right|=20log\left|\frac{FFT\left(S_{FE-Tx}\right)}{FFT{\left(S_{ref}\right)}^{1/2}}\right|-cte;$$

The magnitude: $20log\left|TF\left(FE_{Tx}\right)\right|+cte$ is defined as: $20log\left|TF{\left(FE_{Tx}\right)}^{*}\right|$. This magnitude is straightforwardly obtained from the measurements and represents the frequency profile of the FE response in Tx mode:

(22) $$20log\left|TF{\left(FE_{Tx}\right)}^{*}\right|=20log\left|\frac{FFT\left(S_{FE-Tx}\right)}{FFT{\left(S_{ref}\right)}^{1/2}}\right|,$$

## 3. Results

### 3.1. FE Response in Air

**Figure 5:**
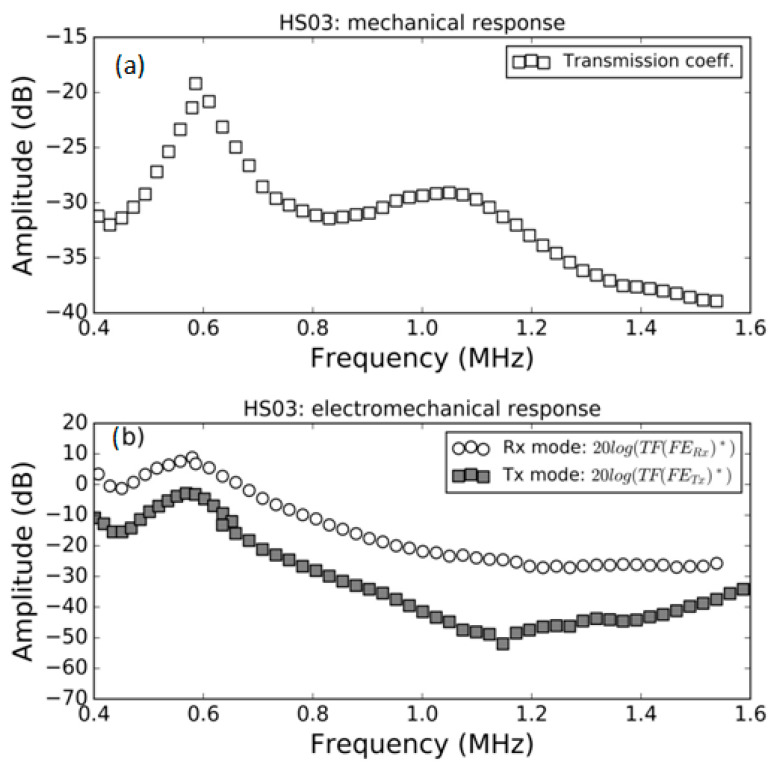
Measured response of the HS03 FE sample in air. (**a**) Modulus of the transmission coefficient vs. frequency (**b**) electromechanical response vs. frequency both in *Tx* and *Rx* mode, (see Equations (16) and (22)).

**Figure 6:**
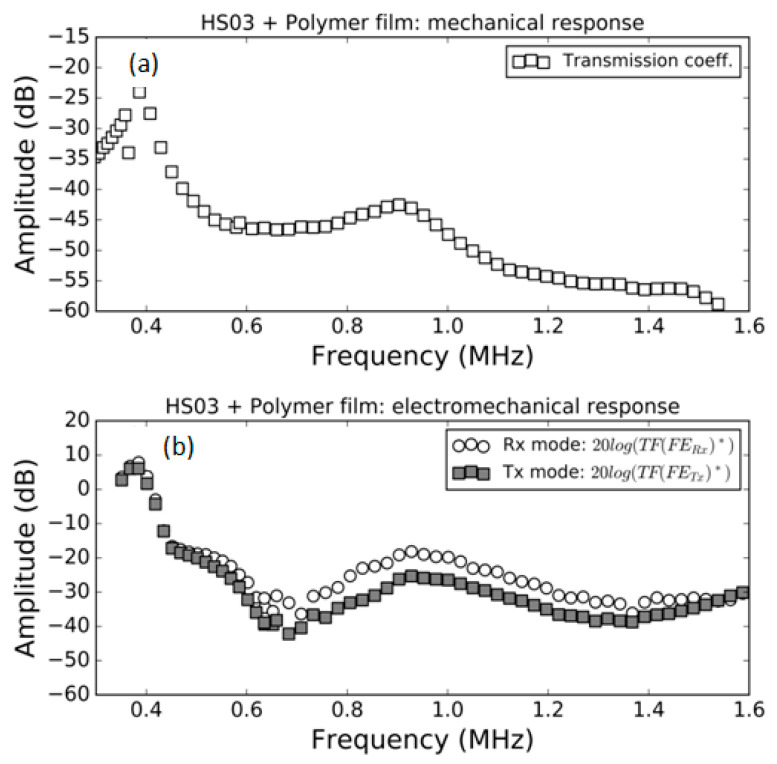
Measured response of the HS03 sample + film in air. (**a**) Modulus of the transmission coefficient vs. frequency; (**b**) electromechanical response vs. frequency both in *Tx* and *Rx* mode, (see Equations (16) and (22)).

**Figure 7:**
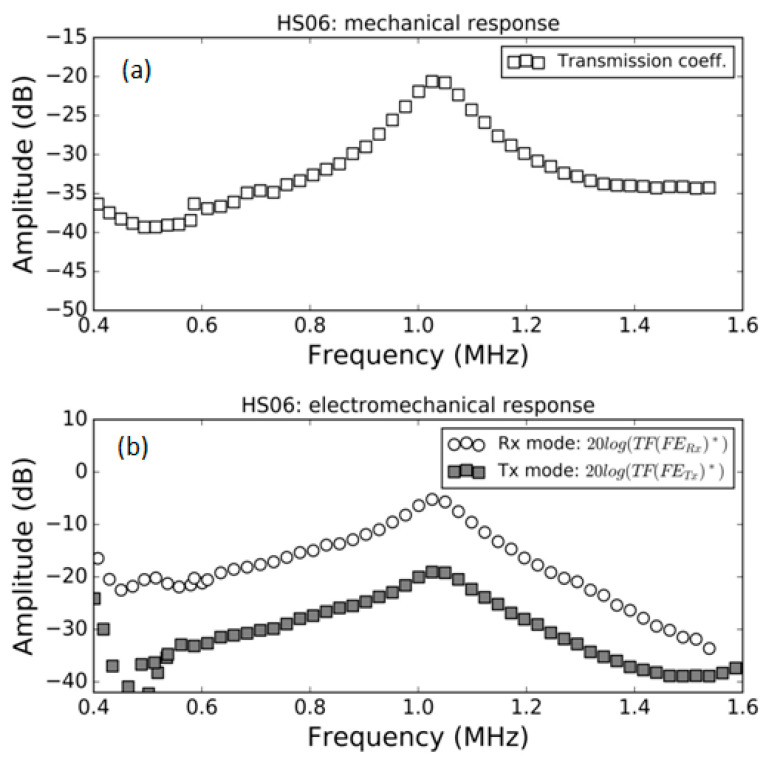
Measured response of the HS06 FE sample in air. (**a**) Modulus of the transmission coefficient vs. frequency; (**b**) electromechanical response vs. frequency both in *Tx* and *Rx* mode, (see Equations (16) and (22)).

Measurement of the modulus of the transmission coefficient and the modulus of the electromechanical response both in *Tx* and *Rx* mode in air for the samples HS03, HS03 + film and HS06 at normal incidence are shown in Figure 5, Figure 6 and Figure 7, respectively. The repeatability of the measurements is typically within the range of the symbol size. Figure 5b, Figure 6b and Figure 7b show, in the same graph, both the Tx and Rx response. This is performed for convenience and there is no reason to expect the same response in Tx and Rx modes. Electromechanical measurements show a larger noise level below −35 dB; this can be attributed to a reduced single to noise ratio. In a similar way, in some cases, a larger dispersion can be found at the limits of the transducer bandwidth; this is also due to a reduced signal-to-noise ratio, in this case produced by the reduced sensitivity of the transducer at the edge of its bandwidth.

The mechanical responses (Figure 5a, Figure 6a and Figure 7a) show the spectra of the transmission coefficient magnitude. These spectra clearly present the effect of the appearance of thickness resonances (located at the frequencies where the transmission coefficient presents a local maximum). Two orders of these resonances are shown in Figure 5a that correspond to the half wavelength resonances (shifted from the theoretically expected value due to the sandwich structure of the film as explained in Ref. [38]). Two orders of these resonances are also shown in Figure 6a. In this case, they correspond, approximately, to the quarter wavelength resonances due to the presence of the adhesive film. Finally, Figure 7a shows the first thickness resonance (half wavelength mode) for the HS06 sample. The electromechanical response follows a similar trend with the only exception of the second order resonance in Figure 5 that presents no electromechanical counterpart. These results are carefully discussed in the Discussion section.

### 3.2. FE Response in Water

**Figure 8:**
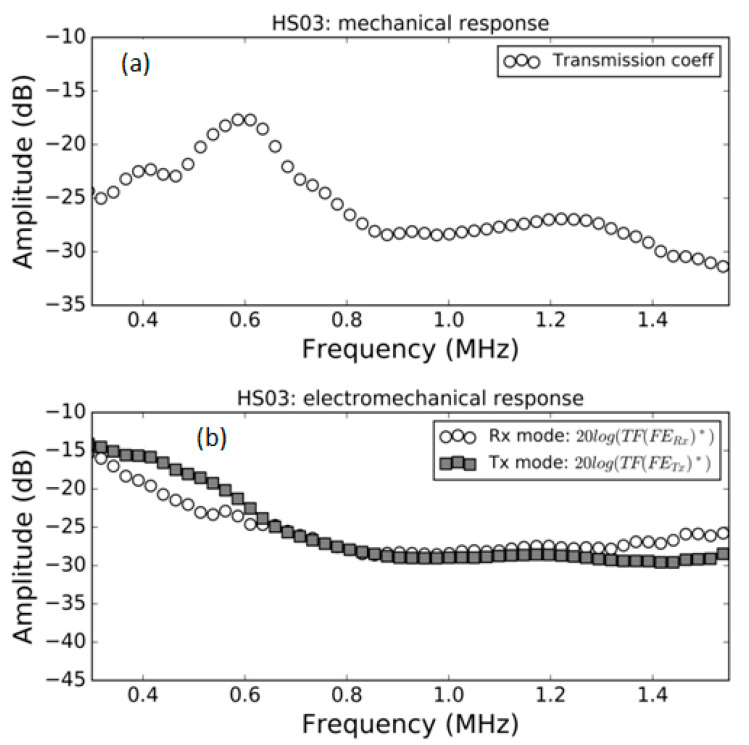
Measured response of the HS03 FE sample in water. (**a**) Modulus of the transmission coefficient vs. frequency; (**b**) electromechanical response vs. frequency both in *Tx* and *Rx* mode, (see Equations (16) and (22)).

**Figure 9:**
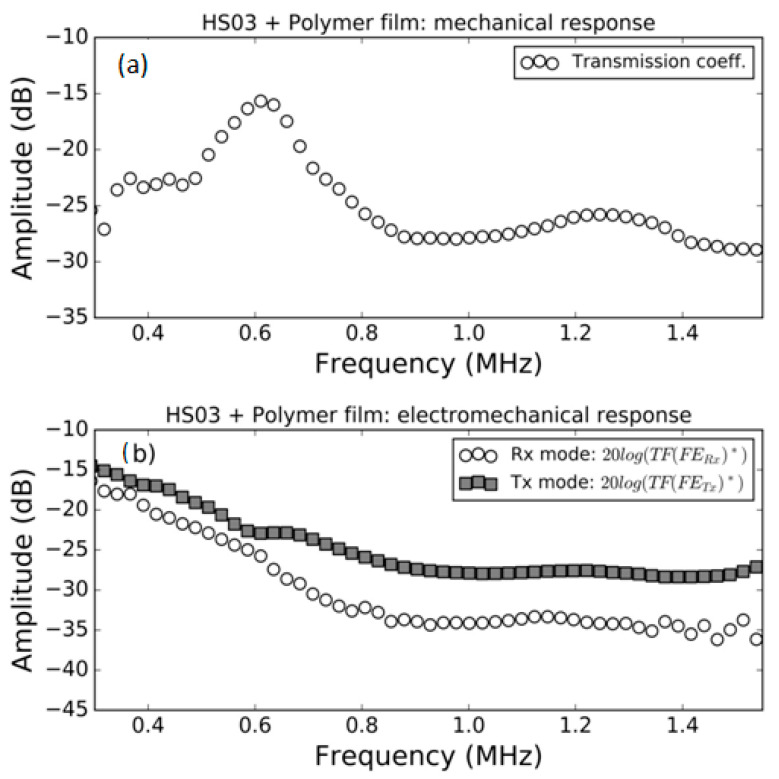
Measured response of the HS03 FE + adhesive film sample in water. (**a**) Modulus of the transmission coefficient vs. frequency; (**b**) electromechanical response vs. frequency both in *Tx* and *Rx* mode, (see Equations (16) and (22)).

**Figure 10:**
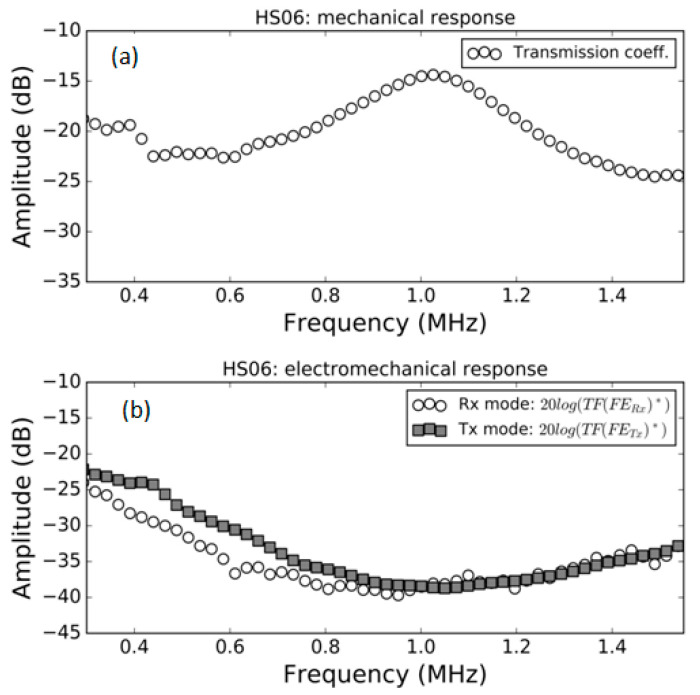
Measured response of the HS06 FE sample in water. (**a**) Modulus of the transmission coefficient vs. frequency; (**b**) electromechanical response vs. frequency both in *Tx* and *Rx* mode, (see Equations (16) and (22)).

Measurements of the modulus of the transmission coefficient and of the electromechanical response both in *Tx* and *Rx* mode in water for the samples HS03, HS03 + film and HS06 at normal incidence are shown in Figure 8, Figure 9 and Figure 10, respectively. The repeatability of the measurements is typically within the range of the symbol size. Figure 8b, Figure 9b and Figure 10b show, in the same graph, both the Tx and Rx response. This is performed for convenience and there is no reason to expect the same response in Tx and Rx modes. Electromechanical measurements show a larger noise level below −35 dB; this can be attributed to a reduced single-to-noise ratio.

The mechanical responses (Figure 8a, Figure 9a and Figure 10a) show the spectra of the transmission coefficient magnitude. These spectra clearly present the effect of the appearance of thickness resonances (where the transmission coefficient presents a local maximum). Two orders of these resonances are shown in Figure 8a that correspond to the half wavelength resonances (shifted due to the sandwich structure of the film, as explained in Ref. [38]. Two orders of these resonances are also shown in Figure 9a. In this case, they also correspond, approximately, to the half wavelength resonances due to the fact that the presence of water eliminates the effect of the adhesive film. Finally, Figure 10a shows the first thickness resonance (half wavelength mode) for the HS06 sample. Unlike in the previous case (air-coupled), the electromechanical response does not follow a similar trend and no electromechanical resonances appear in this case. These results are carefully discussed in the Discussion section.

## 4. Discussion

### 4.1. Discussion of the Modification of the FE Mechanical Response for Two Different External Loads: Air and Water

The mechanical response of the FE samples is studied through the analysis of the magnitude spectrum of the transmission coefficient for ultrasonic waves measured at normal incidence and in a frequency range that includes, at least, the first order thickness resonance of the FE film.

Transmission coefficient measurements of these films in air have been previously studied and reported (see Refs. [15,42]). The only difference in this case, compared with previously published results, is the presence of a sputtered Au layer. The results are shown in Figure 5a, Figure 6a and Figure 7a. The first thickness resonance appears at 0.59 and 1.03 MHz for HS03 and HS06, respectively. These values are slightly smaller than those previously reported (see Table 1). This is due to the presence of the sputtered Au layer. The presence of the adhesive tape film in the HS03 + film sample introduces as much larger load (compared with the load due to the Au layer). As consequence, the displacement towards lower frequencies and lower magnitude values is larger in this case, with the film response approaching a quarter wavelength thickness resonance response. This is similar to what was observed in Ref. [15] when a double-sided electrically conductive adhesive tape was attached to one of the FE film surfaces.

The spectra of the transmission coefficient of the FE samples are modified when water is used instead of air as the outer medium. For the HS03 sample (Figure 5a and Figure 8a), the most significant change is the displacement of the second-order resonance from 1.05 MHz (in air) to 1.25 MHz (in water). In addition, resonances in air are sharper and the transmission coefficient level is, in general, lower. These latter modifications can be explained by the larger impedance mismatch between FE sample and external fluid in the case of air-coupled measurements, but the former modification is quite counterintuitive. Moreover, the harmonic distortion observed in air (with the first order thickness resonance appear at 0.59 MHz and the second one at 1.05 MHz) is almost lost in water (first thickness resonance at 0.6 MHz and second at 1.25 MHz).

As expected, the influence of the adhesive tape film in the HS03 + film sample is almost negligible in water and measurements in water of the transmission coefficient of the HS03 sample (Figure 8a) and the HS03 + film sample (Figure 9a) are almost identical.

The transmission coefficient magnitude in HS06 measured in air is sharper and the overall level is lower compared with the measurements in water. As before, this can be explained by the larger impedance mismatch between the sample and the external fluid when this fluid is air. On the contrary no significant displacement of the resonant frequency is observed.

The modifications observed in the transmission coefficient when the air is replaced with water put forward the question of whether all the observed modifications can be fully explained by the change of the external fluid or if, on the contrary, the FE film undergoes any additional modification in its behavior. This is of interest especially for the HS03 sample where the displacement of the second order resonance towards higher frequency values when the air is replaced with water is difficult to explain by the mere action of the water load.

As this is of interest for this work, and for the potential use of these films for medical transducers and hydrophones, a more detailed analysis of this point has been performed. In particular, the studied films in [42], the same that we have used to fabricate the samples for this work, were used to measure transmission coefficient in water. For these samples, the transmission coefficient in air is well described by a theoretical model based on a layered structure, in particular, a sandwich structure. We have measured transmission coefficient measurements for these samples (HS03 and HS06), but in this case, in water. Then, we have used the same material parameters obtained in [42], from the air-coupled measurements, and used them to calculate the expected response in water. If the FE film remains unmodified, then the calculated transmission coefficient of the film in water using the material parameters obtained from air-coupled measurements should match the experimental measurements. If there is any difference, it can be concluded that the film response is modified when it is immersed in water.

**Figure 11:**
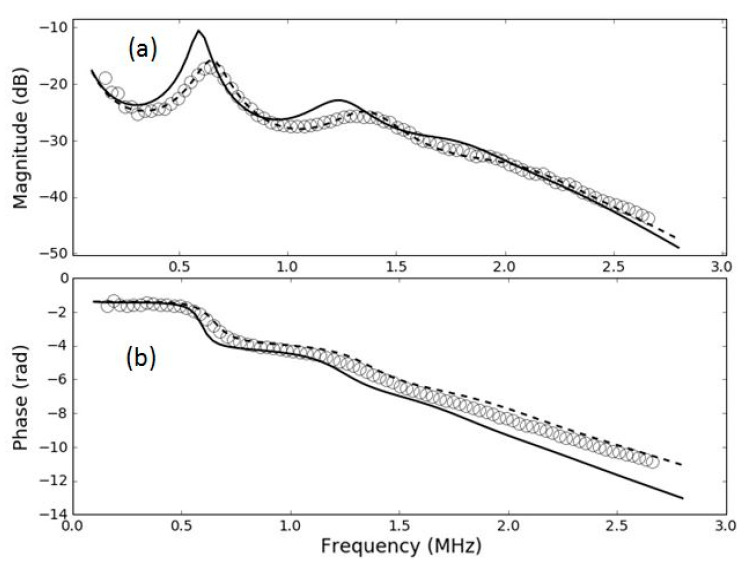
Magnitude (**a**) and phase (**b**) spectra of the transmission coefficient of the HS03 film in water immersion at normal incidence. Open circles: experimental data. Solid line: calculated response using film parameters obtained in [42]. Dashed line: calculated response using film parameters extracted from water immersion measurements.

**Figure 12:**
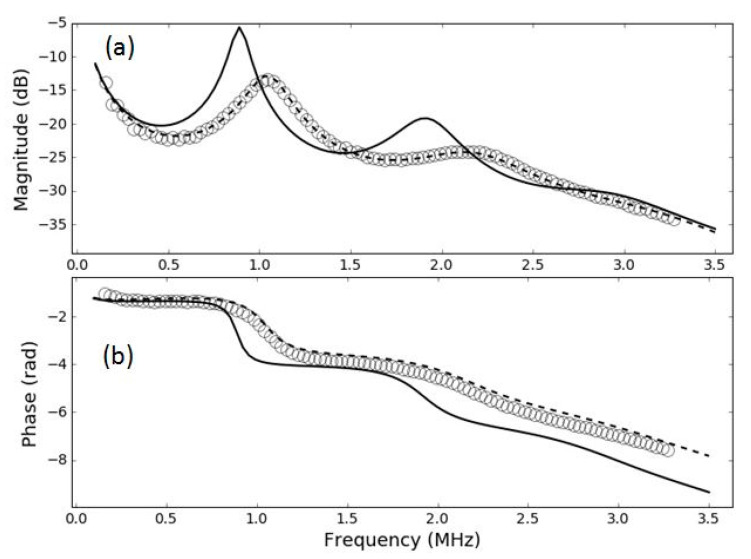
Magnitude (**a**) and phase (**b**) spectra of the transmission coefficient of the HS06 film in water immersion at normal incidence. Open circles: experimental data. Solid line: calculated response using film parameters obtained in [42]. Dashed line: calculated response using film parameters extracted from water immersion measurements.

Results are shown in Figure 11 and Figure 12. These figures show the measured transmission coefficient in water—in this case, both magnitude and phase (open circles)—and the calculated transmission coefficient spectra in water assuming the FE film parameters obtained from measurements in air [42] (solid black line). It is clear that this calculated transmission coefficient fails to explain the measured response in water, so this fact supports the hypothesis that the film itself is modified when it is immersed in water. In addition, the figure also shows the prediction of the sandwich model when material parameters are recalculated for water (using the same procedure as in [42])—this is the dashed line. Clearly, the sandwich model is still able to reproduce the measured response in water, but the material parameters have to be changed. It can be seen from Figure 11 and Figure 12 that this modification of the FE film is larger for the HS06 sample.

One remarkable feature is that in both cases the measured resonances in water appear are higher frequencies compared with the prediction obtained using the film parameters obtained from the air-coupled measurements and using water as outer medium.

It was verified that after water immersion films response in air-coupled measurements are the same as before immersion, without the need of any recovery time, so the mechanism for this modification must be reversible and operates without any delay. This together with the fact that FE surface is impervious support the hypothesis that this modification is not due to water percolation. Moreover, it was observed that the response in water is similar when other fluids are used instead of water (e.g., sunflower oil), so this discards any potential effect of the polar character of the water. In a similar way, as the sample is only submerged a few mm, the effect of hydrostatic pressure on the film must also be discarded.

### 4.2. Electromechanical Response of the FE Films in Air and in Water

Unlike differences in the transmission coefficient, which required of a very detailed analysis to reveal the actual modification of the FE film response when the external fluid is changed (from air to water), the differences in the electromechanical response are evident.

In general, the variation with the frequency in the electromechanical response (both in *Tx* and *Rx* mode) in air follows the observed variation in the transmission coefficient. This is an expected result as at the resonant frequency of the film thickness mode, the strain and stress in the film is maximum due to the additive contribution of the reverberations within the film; therefore, it can be expected that the electromechanical conversion is also maximal at resonant frequencies. This response is observed in all cases, with the only exception being the second order resonance in the HS03 sample (Figure 5).

However, this behavior is completely different for samples in water. Thickness resonances of the FE samples are still present when the FE films are put in water (as can be seen Figure 8a, Figure 9a and Figure 10a), and this is a fully expected result given the large impedance difference between the FE films and the water. However, the electromechanical response does not follow the same trend as the transmission coefficient and the onset of mechanical resonances has no counterpart on the electromechanical response either in transmission or in reception mode.

**Figure 13:**
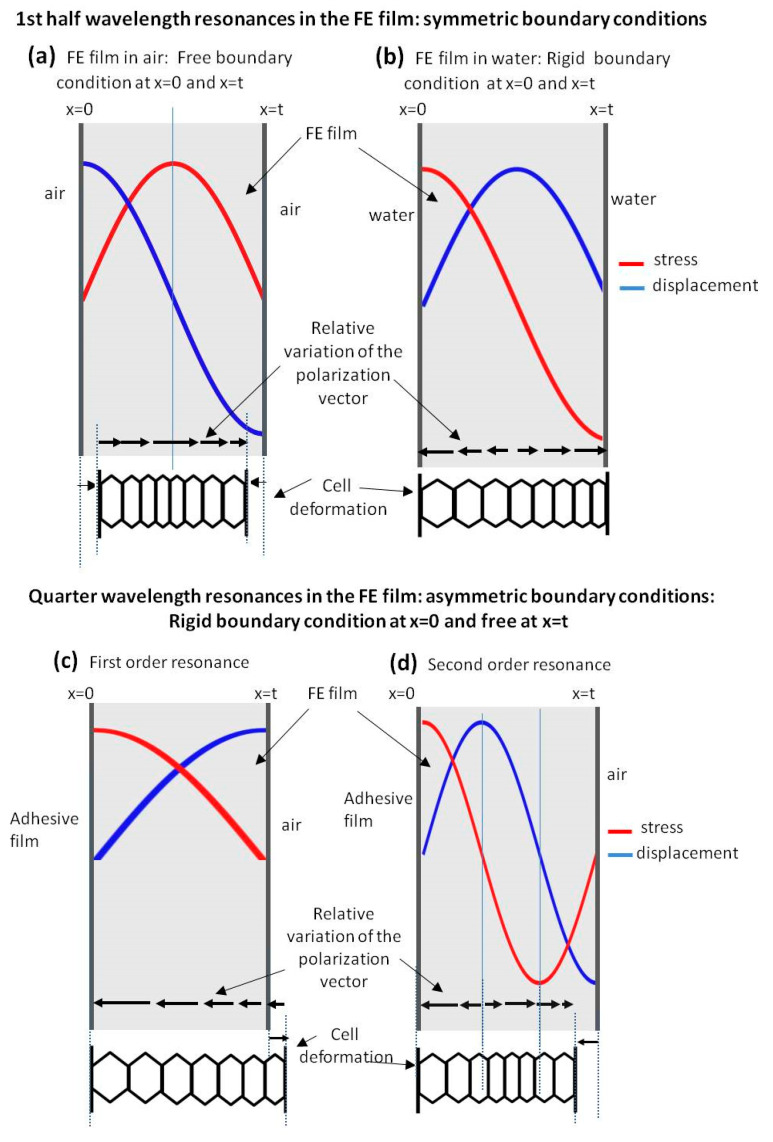
Representation of strain and stress, polarization variation and cell deformation distribution along the film thickness for different boundary conditions and resonance orders; (**a**) first half wave resonance with free boundary conditions; (**b**) first half wave resonance with rigid boundary conditions; (**c**) first quarter wavelength resonance; (**d**) second order quarter wave resonance.

This can be attributed to the different nature of the boundary conditions in both cases (air and water) and the different modification of the polarization inside the material due to the resonances in the film. The situation is schematically explained in Figure 13.

Under air load, the impedance of the film is about 100 times larger than the impedance of the outer medium (air). Then, it can be assumed that the boundary conditions at the FE film surface are very close to those of a free boundary, i.e., maximal displacement and null stress. On the other hand, under water load, the impedance of the outer medium (water) is about 38 times the impedance of the film. Then, the boundary conditions at the FE film surface for the water-loaded case can be assumed to be very close to the rigid boundary condition, i.e., maximum stress and null displacement. Figure 13 schematically represents these two situations and the different pressure and displacement distribution across the film thickness produced by these different boundary conditions. In addition, cell deformation along the thickness is also depicted as well as the relative variation of the polarization vector.

As it can be seen in Figure 13a, the air-loaded case, the modification of the polarization in the FE cells is maximal at the center of the film and minimal on the surface, and the sign of the modification of the polarization vector is the same along the whole film thickness. Therefore, this results in a net variation of the mean polarization in the film. On the other hand, for the water-loaded case, the relative variation of the polarization inside the FE film has opposite signs in the two halves of the film (while polarization keeps the same direction in all the film in some part of the film it increases while in the other it decreases); therefore, it can be expected that the overall polarization modification is null. This explains the lack of electromechanical resonances in the water-loaded films.

The reason of the lack of the second order electromechanical resonance in Figure 5b is the same one that explains the lack of even piezoelectric thickness resonances in a piezoelectric plate and the reasoning is similar to that given for Figure 13b.

Quarter wavelength resonances are observed in the case of the film + adhesive tape in air (Figure 6b) or in the well-known case of air-coupled FE transducers (with a heavy backing). This resonant mode (Figure 6b) does present electromechanical resonances in both the first and the second order thickness resonances. This is explained in Figure 13c,d, where it is shown that under these conditions the net polarization modification along the FE film thickness is not null.

## 5. Conclusions

This work shows that the response of thickness resonances and their associated electromechanical response in FE films is different in water and in air. The impedance of water is much larger than the impedance of the FE film; for this reason, boundary conditions at the FE surface are close to ideal rigid when the FE film is in water. This gives rise to a stress, displacement and polarization change distribution along the film thickness where the overall polarization modification is close to zero. On the contrary, when the film is in air, the impedance of the air is much lower than the impedance of the film and the boundary conditions are close to that of a free surface. Under these conditions, and for the uneven thickness resonance orders, the stress, displacement and polarization distribution along the film thickness gives rise to a net polarization variation. FE films under asymmetric conditions (quarter wavelength resonances) are close to this latter case, with the main difference that electromechanical resonances are observed for all orders of the mechanical resonances.

This difference in the ability of the film to couple mechanical into electrical energy, depending on the external fluid (that is on the boundary conditions), can also be the reason for the subtle differences observed in the transmission coefficient spectra.

## Figures and Tables

**Table 1:** Properties of the FE films employed [10].

Material	Thickness (μm)	Density (kg/m^3^)	λ/2 Resonant Frequency, Thickness Mode (MHz)	Impedance (MRayl)
HS03	70	530	0.638	0.046
HS06	90	370	1.120	0.065

## Data Availability

The data presented in this study are available on request from the corresponding author and will also be publicly available in www.us-biomat.com (accessed on 29 August 2021).

## References

[1] Savolainen A., Kirjavainen K. (1989). Electrothermomechanical film. Part I. Design and characteristics. J. Macromol. Sci. A-Chem..

[2] Sessler G.M., Hillenbrand J. (1999). Electromechanical response of cellular electret films. Appl. Phys. Lett..

[3] Gibson L., Ashby M.F., Brendan A.H. (2010). Cellular Materials in Nature and Medicine.

[4] Restrepo D., Mankame N.D., Zavattieri P.D. (2016). Programmable materials based on periodic cellular solids. Part I: Experiments. Int. J. Solids Struct..

[5] Wegener M., Wirges W., Fohlmeister J., Tiersch B., Gerhard-Multhaupt R. (2004). Two-step inflation of cellular polypropylene films: Void-thickness increase and enhanced electromechanical properties. J. Phys. D Appl. Phys..

[6] Altafim R.A.P., Qiu X., Wirges W., Gerhard R., Basso H.C., Jenninger W., Wagner J. (2009). Template-based fluoroethylenepropylene piezoelectrets with tubular channels for transducer applications. J. Appl. Phys..

[7] Zhang X., Sessler G.M., Wang Y. (2014). Fluoroethylenepropylene ferroelectret films with cross-tunnel structure for piezoelectric transducers and micro energy harvesters. J. Appl. Phys..

[8] Dali O.B., Zhukov S., Rutsch M., Hartman C., von Seggern H., Sessler G.M., Kupnik M. Biodegradable additive manufactured ferroelectret ultrasonic transducer with large output pressure. Proceedings of the IEEE International Ulrasonic Symposium.

[9] Qiu X., Wegener M., Wirges W., Zhang X., Hillenbrand J., Xia Z., Gerhard-Multhaupt R., Sessler G.M. (2005). Penetration of sulfur hexafluoride into cellular polypropylene films and its effect on the electric charging and electromechanical response of ferroelectrets. J. Phys. D Appl. Phys..

[10] Wegener M., Wirges W., Gerhard-Multhaupt R., Bauer-Gogonea S., Paajanen M., Minkkinen H., Raukola J., Dansachmüller M., Schwodiauer R., Bauer S. (2004). Controlled inflation of voids in cellular polymer ferroelectrets: Optimizing electromechanical transducer properties. Appl. Phys. Lett..

[11] Mellinger A., Wegener M., Wirges W., Mallepally R.R., Gerhard-Multhaupt R. (2006). Thermal and Temporal Stability of Ferroelectret Films Made from Cellular Polypropylene/Air Composites. Ferroelectrics.

[12] Saarimaki E., Paajanen M., Savijarvi A., Minkkinen H., Wegener M., Voronina O., Schulze R., Wirges W., Gerhard-Multhaupt R. (2006). Novel heat durable electromechanical film: Processing for electromechanical and electret applications. IEEE Trans. Dielectr. Electr. Insul..

[13] Qiu X., Mellinger A., Wegener M., Wirges W., Gerhard R. (2007). Barrier discharges in cellular polypropylene ferroelectrets: How do they influence the electromechanical properties?. J. Appl. Phys..

[14] Fang P., Wirges W., Wegener M., Zirkel L., Gerhard R. (2008). Cellular polyethylene-naphthalate films for ferroelectret applications: Foaming, inflation and stretching, assessment of electromechanically relevant structural features. e-Polymers.

[15] Álvarez-Arenas T.E.G., Calás H., Cuello J.E., Fernández A.R., Muñoz M. (2010). Noncontact ultrasonic spectroscopy applied to the study of polypropylene ferroelectrets. J. Appl. Phys..

[16] Mohebbi A., Mighri F., Ajji A., Rodrigue D. (2018). Cellular Polymer Ferroelectret: A Review on Their Development and Their Piezoelectric Properties. Adv. Polym. Technol..

[17] Liu B., Han L., Pan L., Li H., Zhao J., Dong Y., Wang X. (2021). Flexible Multiscale Pore Hybrid Self-Powered Sensor for Heart Sound Detection. Sensors.

[18] Sessler G.M., Hillenbrand J. Novel Silicon and Polymer Sensors in Acoustics. Proceedings of the SENSOR+TEST Conference.

[19] Luo Z., Shi J., Beeby S.P. (2016). Novel thick-foam ferroelectret with engineered voids for energy harvesting applications. J. Phys. Conf. Ser..

[20] Zhang Y., Bowen C.R., Ghosh S.K., Mandal D., Khanbareh H., Arafa M., Wan C. (2019). Ferroelectret materials and devices for energy harvesting applications. Nano Energy.

[21] Zhang X., Pondrom P., Sessler G.M., Ma X. (2018). Ferroelectret nanogenerator with large transverse piezoelectric activity. Nano Energy.

[22] Ma X., Zhang X., Fang P. (2017). Flexible film-transducers based on polypropylene piezoelectrets: Fabrication, properties, and applications in wearable devices. Sens. Actuators A Phys..

[23] Shi J., Beeby S. Textile based ferroelectret for foot pressure sensor. Proceedings of the IEEE International Conference on Flexible and Printable Sensors and Systems (FLEPS).

[24] Buchberger G., Schwödiauer R., Arnold N., Bauer S. (2008). Cellular ferroelectrets for flexible touchpads, keyboards and tactile sensors. Proc. IEEE Sens..

[25] Sborikas M., Wegener M. (2013). Cellular-foam polypropylene ferroelectrets with increased film thickness and reduced resonance frequency. Appl. Phys. Lett..

[26] Kressmann R. (2001). New piezoelectric polymer for air-borne and water-borne sound transducers. J. Acoust. Soc. Am..

[27] Wegener M., Tuncer E., Wirges W., Gerhard R., Dansachmuller M., Bauergogonea S., Schwodiauer R., Bauer S. (2004). Ferroelectrets: Highly anisotropic electrically charged polymer foams for electromechanical transducer applications. Proc. IEEE Ultrason. Symp..

[28] Döring J., Bartusch J., Beck U., Erhard A. EMFIT ferroelectret film transducers for non-contact ultrasonic testing. Proceedings of the European Conference NDT.

[29] Ealo J., Seco F., Jimenez A. (2008). Broadband EMFi-based transducers for ultrasonic air applications. IEEE Trans. Ultrason. Ferroelectr. Freq. Control.

[30] Gaal M., Bartusch J., Doring J., Dohse E., Lange T., Hillger W., Kreutzbruck M. Air-coupled ferroelectret ultrasonic transducers applied to testing of fiber-reinforced polymers. Proceedings of the International Conference of the Slovenian Society for Non-Destructive Testing.

[31] Álvarez-Arenas T.E.G. (2013). Air-coupled piezoelectric transducers with active polypropylene foam matching layers. Sensors.

[32] Gaal M., Bartusch J., Dohse E., Schadow F., Köppe E. (2016). Focusing of ferroelectret air-coupled ultrasound transducers. AIP Conf..

[33] Tang J., Tong L., Xiang Y., Qiu X., Deng M., Xuan F. (2019). Design, fabrication and characterization of Emfi-based ferroelectret air-coupled ultrasonic transducer. Sens. Actuators A Phys..

[34] Döring J., Bovtun V., Gaal M., Bartusch J., Erhard A., Kreutzbruck M., Yakymenko Y. (2012). Piezoelectric and electrostrictive effects in ferroelectret ultrasonic transducers. J. Appl. Phys..

[35] Bovtun V., Döring J., Bartusch J., Gaal M., Erhard A., Kreutzbruck M. (2013). Enhanced electromechanical response of ferroelectret ultrasonic transducers under high voltage excitation. Adv. Appl. Ceram..

[36] De Medeiros L.J., Kamimura H.A.S., Altafim R.A.P., Carneiro A.A.O., Amorim M.F., Altafim R.A.C. (2015). Piezoelectret-based hydrophone: An alternative device for vibro-acoustography. Meas. Sci. Technol..

[37] Palitó T.T.C., Assagra Y.A.O., Altafim R.A.P., Carmo J.P., Altafim R.A.C. (2019). Low-cost electro-acoustic system based on ferroelectret transducer for characterizing liquids. Meas. J. Int. Meas. Confed..

[38] De Luna D.R., Palitó T., Assagra Y., Altafim R., Carmo J., Carneiro A., de Sousa J.V.A. (2020). Ferroelectret-based hydrophone employed in oil identification-A machine learning approach. Sensors.

[39] Álvarez-Arenas T.E.G., Diez L. Ferroelectret transducers for water immersion and medical imaging. Proceedings of the IEEE International Ultrasonic Symposium (IUS).

[40] Aguilar J.Q., Álvarez-Arenas T.E.G. Optimization of ferroelectret transducers for pulse-echo water immersion operation. Proceedings of the IEEE International Ultrasonics Symposium (IUS).

[41] Aguilar J.Q., Svilainis L., Camacho J., Álvarez-Arenas T.E.G. (2020). Ferroelectret Ultrasonic Transducers for Pulse-Echo Water Immersion. Appl. Sci..

[42] Aguilar J.Q., Munoz M., Álvarez-Arenas T.E.G. (2021). Interpretation of the Thickness Resonances in Ferroelectret Films Based on a Layered Sandwich Mesostructure and a Cellular Microstructure. IEEE Trans. Ultrason. Ferroelectr. Freq. Control.

